# Two diphtheria sub-clusters with autochthonous cases in Germany and Poland within a *Corynebacterium diphtheriae* ST-574 outbreak, 2022 to July 2025

**DOI:** 10.2807/1560-7917.ES.2025.30.33.2500539

**Published:** 2025-08-21

**Authors:** Imme Friederike Roewer de Porto, Alexandra Dangel, Lena Schneider, Aleksandra A. Zasada, Jonas Haller, Inas Abdelgawad, Claudia Siffczyk, Iwona Paradowska-Stankiewicz, Ole Wichmann, Franziska Badenschier, Anja Berger, Cornelius Rau, Andreas Sing

**Affiliations:** 1 Department for Infectious Disease Epidemiology, Robert Koch Institute, Berlin, Germany; 2 Postgraduate Training for Applied Epidemiology, Department for Infectious Disease Epidemiology, Robert Koch Institute, Berlin, Germany; 3 ECDC Fellowship Programme, Field Epidemiology path (EPIET), European Centre for Disease Prevention and Control (ECDC), Stockholm, Sweden; 4 NGS-Core Unit, Bavarian Health and Food Safety Authority, Oberschleißheim, Germany; 5 Department of Sera and Vaccines Evaluation, National Institute of Public Health NIH – National Research Institute, Warsaw, Poland; 6 Gesundheitsamt Frankfurt am Main, Frankfurt am Main, Germany; 7 Local Public Health Authority Havelland, Brandenburg, Germany; 8 Forensic Medicine and Clinical Toxicology Department, Faculty of Medicine, Cairo University, Al Kasr Al Aini, Cairo, Cairo Governorate, Egypt; 9 Department of Infectious Diseases Epidemiology and Surveillance, National Institute of Public Health NIH – National Research Institute, Warsaw, Poland; 10 National Consiliary Laboratory for Diphtheria, Bavarian Health and Food Safety Authority, Oberschleißheim, Germany; 11 EU Public Health Reference Laboratory for Diphtheria and Pertussis (EURL-PH-EUDIPE), Oberschleißheim, Germany; 12 WHO Collaborating Centre for Diphtheria, Oberschleißheim, Germany; * These authors contributed equally to this work and share first/last authorship.

**Keywords:** ST-574, Multilocus Sequence Typing, Corynebacterium diphtheriae, Diphtheria, Disease outbreaks, Migration and health, Next Generation Sequencing, Outbreak investigation, Vaccine-preventable disease

## Abstract

Within a *Corynebacterium diphtheriae* ST-574 outbreak comprising mostly migration-associated cases since 2022, Germany detected two sub-clusters in 2025. Among sub-cluster cases with travel information (24/26), 22 were acquired autochthonously. One of two imported cases came from a Polish voivodeship having an additional case. Sub-cluster 1 included 16 cutaneous cases, sub-cluster 2 included six cutaneous and five respiratory cases. One unvaccinated child and two older adults died. Of 27 cases, 12 experienced homelessness. Strengthened immunisation, improved wound care and international surveillance are needed.

In February 2025, whole-genome sequencing (WGS) at Germany’s National Conciliary Laboratory for Diphtheria (NCLD) identified two sub-clusters of diphtheria with *Corynebacterium diphtheriae* belonging to the sequence type 574 (ST-574). The two sub-clusters had evolved from a larger ST-574 cluster originally imported in 2022 [1,2]. Among the cases detected in Germany belonging to these two sub-clusters, 13 of 16 cases in sub-cluster 1 and nine of 10 cases in sub-cluster 2 had neither travel nor a migration history within 21 days before symptom onset, suggesting ongoing autochthonous transmission and triggering an outbreak investigation. One case in sub-cluster 2 had been imported to Germany from a voivodeship in Poland, where an additional case was identified [3]; the isolate of the latter case was subsequently characterised as belonging to sub-cluster 2. Collaboration between Polish and German laboratories for diphtheria diagnostics was initiated to uncover genomic links between cases in both countries followed by cooperation between German and Polish national public health institutes to complete additional epidemiological information for the case detected in Poland.

We describe all confirmed ST-574 outbreak cases in Germany and Poland between 01 January 2022 and 03 July 2025, focusing on the two sub-clusters, and presenting their epidemiological and genomic characteristics.

## Outbreak investigation and case definitions

Detection of *Corynebacterium* spp. bearing a diphtheria toxin gene (*tox*) and/or producing diphtheria toxin (DT) is notifiable according to the German Infection Protection Act (IfSG) [4] and the Polish Act on the Prevention and Combating of Infections and Infectious Diseases in Humans (Dz. U. 2008 Nr 234 poz. 1570) [5]. We included all patients reported in Germany and Poland between 01 January 2022 and 03 July 2025 with isolates that either produced DT or carried the *tox* gene (non-toxigenic *tox*-gene bearing, NTTB), irrespective of the DT detection via the Elek test [6] or lateral flow immunoassay result [7]. Isolates that carry the *tox* gene but do not produce detectable DT in the laboratory remain epidemiologically relevant because they can regain DT production in vivo and indicate the circulation of *tox*-positive strains. We matched these isolates to all notified cases. 

We defined a confirmed case as a patient whose *C. diphtheriae* isolate was identified by matrix-assisted laser desorption ionisation-time of flight mass spectroscopy (MALDI-TOF MS; MALDI Biotyper; Bruker Daltonics, Bremen, Germany) [8], and then assigned to ST-574 by multilocus sequence typing (MLST). A probable case was any case with a *tox*-PCR-positive isolate that showed sole resistance to trimethoprim/sulfamethoxazole (cotrimoxazole), which was defined according to European Committee on Antimicrobial Susceptibility Testing (EUCAST) guidelines [9], a characteristic observed in every ST-574 isolate, or any case with *C. diphtheriae* linked epidemiologically to a confirmed case. A suspected case was any newly notified *C. diphtheriae* infection that lacked typing or resistance testing results.

All 125 isolates collected in Germany as well as the single Polish isolate, which was entered at the library-preparation step, were subjected to WGS. Procedures for WGS wetlab workflows, MLST, core genome (cg) MLST and minimum spanning tree (MST) generation were performed as reported in Berger et al. [10]. Additional analyses such as a SNP-phylogeny and prediction of resistance and virulence genes are described in the Supplementary Material. In addition to case information already provided in statutory surveillance, local public health authorities in Germany and Poland collected epidemiological data such as housing status and substance use. Imported cases were defined as cases with travel or migration history outside of the country of notification within the 21 days (> twice the maximum incubation period) before symptom onset.

## Epidemiological findings and case characteristics

**Figure 1:**
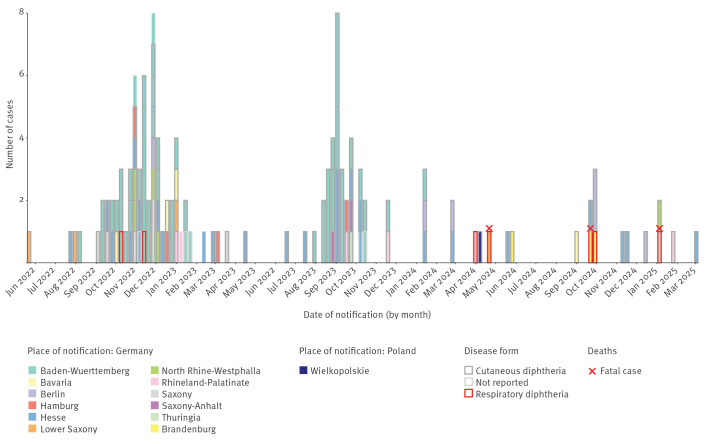
Number of diphtheria ST-574 confirmed cases in Germany (n = 125) and Poland (n = 1) by federal state or voivodeship, manifestation and clinical outcome, 01 January 2022–03 July 2025

We identified 126 confirmed (Germany 125, Poland 1) and four probable cases (all in Germany). Two waves of cases respectively occurred in autumn/winter 2022 and 2023 and were followed by scattered case notifications in 2024 and 2025 (2022: 54; 2023: 49; 2024: 19; 2025: 4 cases; Figure 1).

Of 126 confirmed cases, 113 (90%) were male, the median age was 19 years, and 101 (80%) cases were imported. Cutaneous diphtheria was most common and observed in 110 cases (87%) (Table 1). Respiratory cases appeared to proportionally increase between 2022 and 2025, with two of 54 cases in 2022, four of 19 cases in 2024 and one of four cases in 2025. In 2023, no respiratory case was reported. Three deaths occurred (case-fatality ratio 2.4% overall with 3 fatal cases among 7 cases with respiratory manifestation). Vaccination status was unknown for 89 (71%) cases (Table 1).

**Table 1:** Characteristics of diphtheria ST-574 confirmed cases in Germany (n = 125) and Poland (n = 1) by sub-cluster, 01 January 2022–03 July 2025

Characteristics	Imported non-sub-cluster (99 cases)		Sub-cluster 1 (16 cases)^a^	Sub-cluster 2 (11 cases)^a^	Total (126 cases)	
	Number^b^	%^a^	Number^b,c^	Number^b,c^	Number^b^	%^b^
Diagnostics						
Only *tox* PCR positive	2	2	9	0	11	9
PCR and Elek test positive	97	98	7	11	115	91
Age						
Median age in years (Q1–Q3)	18 (16–21)		45 (39–55)	58 (48–64)	19 (17–27)	
Administrative sex and gender						
Female	0	0	3	3	6	5
Male	93	94	12	8	113	90
Gender-diverse	1	1	0	0	1	1
Unknown	5	5	1	0	6	5
Manifestation						
Respiratory diphtheria	2	2	0	5	7	6
Cutaneous diphtheria	88	89	16	6	110	87
Unknown	9	9	0	0	9	7
Severity						
Hospitalised	25	25	8	11	44	35
Median hospital stay in days (Q1–Q3)	5 (0–8)		1 (0–6.5)	1 (0–12)	2 (0–8)	
Death due to disease	0	0	0	3	3	2
Vaccination status						
Vaccination unknown	69	70	11	9	89	71
Unvaccinated	20	20	2	1	23	18
Vaccinated (ever, at least once)	10	10	3	1	14	11

Q: quartile.

^a^ Sub-cluster 1 comprises 16 cases including 13 autochthonous in Germany, one imported from outside of Europe and two for which the country of infection is unknown; sub-cluster 2 comprises 11 cases including nine autochthonous in Germany, one autochthonous in Poland, and one imported to Germany from Poland.

^b^ The content of the cells corresponds to what is described in the column heading, unless this is otherwise specified by a row heading.

^c^ Percentages are not presented when the total number of cases is less than 60.

Sub-cluster 1 (≤ 8 allelic differences;  ≤ 17 SNPs; 16 cases from June 2023 to May 2025) comprised 12 male cases, three female cases and one for which sex was unknown (Table 1). The median age in this sub-cluster was 45 years and among the 16 cases, 13 cases were autochthonous in Germany. All 16 cases in the sub-cluster presented as cutaneous diphtheria with nine isolates being NTTB (2023:1/4; 2024: 5/9; 2025:3/3). Twelve of the sub-cluster 1 cases were primarily concentrated in central Germany near Frankfurt am Main, Hesse (further referred to as Frankfurt), with 11 notified in Hesse and one in Rhineland-Palatinate. The remaining four cases were notified in four different federal states (Baden-Württemberg, Bavaria, Brandenburg and North Rhine-Westphalia). Of these four, three cases reported having been to Frankfurt, Hesse, in the 21 days before disease onset: one person travelled to Frankfurt to consume drugs, another was working in Frankfurt and a third travelled through Frankfurt central station. Among all sub-cluster 1 cases, 12 in total reported either living or staying/travelling in Frankfurt. Six individuals were experiencing homelessness, with three of them using emergency shelters. Four cases including one case not living in Frankfurt had contact with people using or selling drugs around Frankfurt central station. One case was classified as imported from outside of Europe with reported travel to a northern African country for a few days within the 21 days before symptom onset. For two cases how diphtheria was acquired was unknown (Table 2).

**Table 2:** Epidemiological case characteristics of sub-clusters of diphtheria ST-574 confirmed cases in Germany and Poland, June 2023–January 2025 (n = 27)

Characteristics	Sub-cluster 1 (16 cases)	Sub-cluster 2 (11 cases)
	Number of cases	Number of cases
Year of notification		
**2023**	**4**	**0**
**2024**	**9**	**10**
**2025**	**3**	**1**
Acquisition		
**Autochthonous in Germany**	**13**	**9**
Staying in or travelling to or through Frankfurt am Main, Hesse	12	0
**Autochthonous in Poland**	**0**	**1**
**Imported**	**1^a^**	**1^b^**
**Unknown**	**2**	**0**
Housing		
**Experiencing homelessness**	**6**	**6**
Shelter for people in homelessness	3	0
No reported use of shelter for people in homelessness	1	5
Unknown	2	1
**Not experiencing homelessness**	**8**	**5**
**Unknown housing status**	**2**	**0**
Substance abuse		
**Reported substance use or alcohol abuse**	**10**	**2**
Reported use of drugs	7	1
Reported use of alcohol (n)	3	1
**No reported substance or alcohol abuse (n)**	**0**	**0**
**Unknown (n)**	**6**	**9**

^a^ Imported from outside of Europe (North Africa).

^b^ Imported from Poland into Germany.

Sub-cluster 2 (≤ 8 allelic differences;  ≤ 12 SNPs; 11 cases from January 2024 to January 2025) comprised eight cases of male sex and three cases of female sex. The median age in the sub-cluster was 45 years and among all cases reported in this sub-cluster, nine cases were autochthonous in Germany and notified there, and one case was autochthonous in Poland and notified in Poland. Among the 10 sub-cluster 2 cases notified in Germany, one was imported from Poland. Of the total 11 cases belonging to sub-cluster 2, six cutaneous diphtheria cases were reported, all among people experiencing homelessness. Five of them were notified in Berlin and one in the Polish voivodeship of Wielkopolskie (Table 2). Five cases presented with respiratory diphtheria. Four of these were notified across three German federal states (Brandenburg, Saxony and Rhineland-Palatinate), two of them in the same household, while one additional case of respiratory diphtheria was imported from Poland. Of cases with respiratory diphtheria, three were fatal: a school-aged child, and two adults, both over 65 years-old, including a care-home resident, and a care worker, who travelled from the Polish voivodeship Wielkopolskie where the other cutaneous case had been notified. No epidemiological link between fatal cases could be established. While the child was unvaccinated, the vaccination status for the two fatal adult cases was unknown.

## Genome sequencing, resistance markers and phylogeny

**Figure 2:**
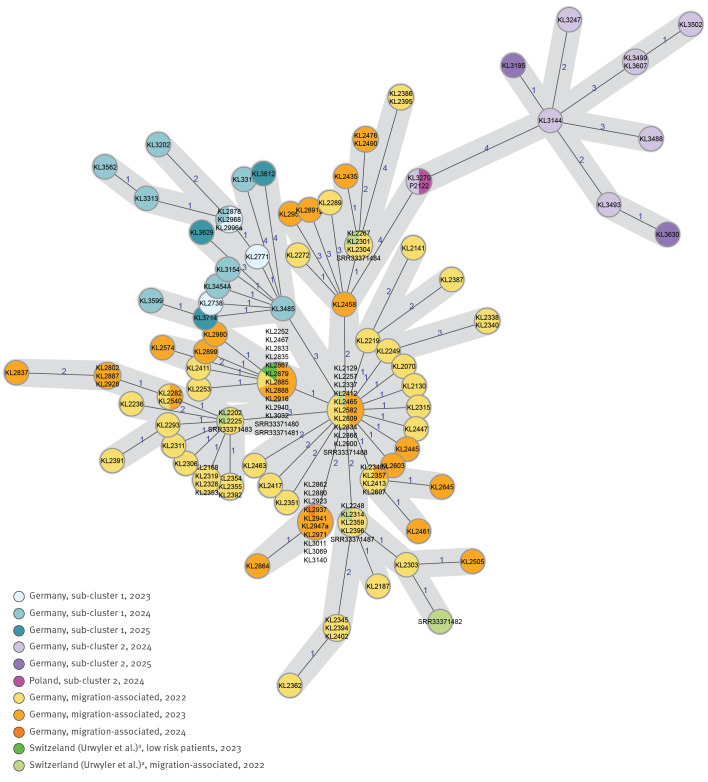
Minimum spanning tree based on core genome multilocus sequence typing analysis of ST-574 *C. diphtheriae* isolates, 2022–2025 (n = 133), from Germany (n = 125), Poland (n = 1) and Switzerland (n = 7)

The genomic sequences assigned to ST-574 by MLST were included in the cgMLST-based MST (Figure 2) as well as in a maximum likelihood (ML) SNP-based phylogeny, with the ML tree depicted in the Supplementary Figure in the Supplementary Material. The cgMLST-based MST shows the two sub-clusters branching off from the main node which primarily consists of isolate genomes of the 2022 outbreak, (Figure 2; blue and purple colours). The SNP-based ML tree confirms the evolving two sub-clusters (Supplementary Figure). Seven ST-574 genomes recently reported from Switzerland were also included as they comprised two autochthonous cases similar to those observed cases in Germany [11]: five from asylum seekers (2022) and two from senior residents (2023). These genomes were not epidemiologically related to the two German sub-clusters but closely phylogenetically related or clonal to isolates obtained from newly-arriving migrants in Germany in 2022 and 2023.

Samples are colour-coded based on combined geographic, sub-cluster and date of detection characteristics of respective cases, and these include 99 isolates of newly-arriving migrants and 27 isolates belonging to either of two sub-clusters identified in the current study: sub-cluster 1 is in blue and sub-cluster 2 is in purple (Germany) and pink (Poland).

All isolates of the ST-574 cluster show a uniform resistance profile including a sole characteristic, which is antimicrobial resistance to trimethoprim/sulfamethoxazole (cotrimoxazole). They also show a uniform genetic profile of detected resistance and virulence genes, as detailed in the Supplementary information on resistance and virulence gene prediction. In addition, for the nine NTTB isolates detected in sub-cluster 1, lack of DT production, determined by Elek test, could be genetically proven by a 11–150 nt truncation of the *tox*-gene at the 5' end, in conjunction with a directly adjacent insertion (IS1132 [12]) to the truncated site.

## Discussion

Since 2022, an outbreak caused by *C. diphtheriae* ST-574 has emerged in Germany following detection among newly-arriving migrants, often seeking asylum [1,2]. Despite no known secondary spread at first, we now observe two separate transmission events within Germany indicating ongoing autochthonous transmission.

Noteworthy, besides newly-arriving migrants, additional vulnerable groups are affected, i.e. people experiencing homelessness, individuals who are using drugs, older adults, and unvaccinated individuals. Importantly, three fatalities due to respiratory diphtheria occurred within the last 12 months, whereas no fatal cases with respiratory diphtheria with *C. diphtheriae* had been recorded in Germany since the beginning of mandatory reporting in 2001. 

All 16 infections in sub-cluster 1 were cutaneous, while sub-cluster 2 included five respiratory cases, three of which were fatal, underlining the potential severity of diphtheria. The first autochthonous diphtheria case in a person experiencing homelessness in Frankfurt detected in February 2023, described by Haller et al. [13], showed severe dual respiratory and cutaneous symptoms. The case met our case definition of a suspected outbreak case since no typing results were available but was not described in more detail as we only described confirmed outbreak cases. Nevertheless, it serves as a reminder that double manifestations can occur.

For all cases except two [10], the transmission chain remains unclear. As multiple cases in both sub-clusters were reported in the same or neighbouring federal states with partial temporal overlap, this suggests unidentified transmissions. In sub-cluster 2, most cases with respiratory disease died, which could indicate that many milder cases of diphtheria are not being detected. Such undetected spread may, e.g. occur in vaccinated individuals with only mild or no symptoms, leaving cases in vulnerable groups as warning sign of wider silent circulation of the pathogen. Awareness in and around Frankfurt was likely higher after first detections and subsequent outbreak investigation in 2023 [13,14]. This highlights the importance of collaborating with low-threshold services to improve access to diagnostics, vaccination and wound care. Interestingly, NTTB cases ST-574 in sub-cluster 1 appear to increase over time which is in line with the documentation of NTTB cases among people experiencing homelessness [15].

Cases of ST-574, mostly among newly-arriving migrants, had also been reported in other Western European countries including Belgium, Switzerland, and the United Kingdom in 2022 [16-18]. While Germany reported most cases, cases linked to Poland suggest wider spread within Europe [3]. In contrast, cases from Switzerland seem to represent secondary cases following importations [3,11]. A Rapid Risk Assessment recently published by the European Centre for Disease Prevention and Control (ECDC) in 2025, also points to a broader circulation of ST-574 across Europe [19]. However, it remains unclear whether autochthonous sub-clusters are unique to ST-574 or could also form in other STs detected during the 2022 outbreak.

The three respiratory diphtheria deaths involved an unvaccinated child and two older adults with unknown vaccination status. Overall, the presence of the NTTB isolates and the homogenous profile of typical *C. diphtheriae* resistance and virulence gene patterns throughout the ST-574 cluster does not indicate signs of increased virulence. Rather, factors such as insufficient or waning immunity, co-morbidities, and delayed care may have contributed to severe outcomes. 

Some limitations of the current study were that vaccination records were often missing, and details on vulnerabilities (e.g. housing, substance use) were difficult to obtain, thus risk factors and immunity gaps remain unclear. Further, contact tracing was not possible in a majority of cases likely leading to an under-detection of secondary cases and epidemiological links. While cases with ST-574 have been reported from other European countries since 2022, aside from the included isolates from Germany, Poland, and Switzerland, no sequence data from other European countries were available.

## Conclusion and recommendations

The rise in ST-574 respiratory diphtheria, associated fatalities, and emergence of two separate clusters in Germany with mostly autochthonous cases, indicate that the situation may have shifted from sporadic importations of diphtheria to increased circulation of the pathogen with ongoing autochthonous transmission. Our findings suggest that countries that observed imported diphtheria cases since 2022 may be susceptible to secondary outbreaks. This investigation demonstrates the value of systematic WGS, active case finding, and international collaboration to uncover ongoing local transmission and link cases across borders. To prevent further cases and deaths, it is crucial to strengthen routine immunisation and close immunity gaps, particularly in vulnerable groups. Protecting these groups requires targeted outreach and close collaboration with low-threshold services so that everyone, everywhere has access to diagnostics, wound care, and vaccination.

## Data Availability

Whole genome sequencing (WGS) data are available under BioProject IDs PRJNA898270, PRJNA1176523, PRJNA1139060, PRJNA1197751, PRJNA1123191 at https://www.ncbi.nlm.nih.gov/bioproject. Detailed accession numbers are given in the Supplemental Table.

## Statements

Imme Roewer de Porto and Lena Schneider are fellows of the German Postgraduate Training in Applied Epidemiology (PAE) associated with the ECDC Fellowship Programme. The views and opinions expressed herein do not state or reflect those of ECDC. ECDC is not responsible for the data and information collation and analysis and cannot be held liable for conclusions or opinions drawn.

## Supplementary Data

401000 Supplementary Material
