# Supplementary material for: Two diphtheria sub-clusters with autochthonous cases in Germany and Poland within a Corynebacterium diphtheriae ST-574 outbreak, 2022 to July 2025
Source: Euro Surveill. 2025 Aug 21;30(33):2500539. doi: 10.2807/1560-7917.ES.2025.30.33.2500539 (PMC12372894; doi:10.2807/1560-7917.ES.2025.30.33.2500539)
Supplement: Supplementary Material [file 25-00539_SupplementaryMaterial.pdf]

**SUPPLEMENTAL MATERIAL**

This supplementary material is hosted by Eurosurveillance as supporting information alongside the article "Two diphtheria sub-clusters with autochthonous cases in Germany and Poland within a Corynebacterium diphtheriae ST-574 outbreak, 2022 - to July 2025", on behalf of the authors, who remain responsible for the accuracy and appropriateness of the content. The same standards for ethics, copyright, attributions and permissions as for the article apply. Supplements are not edited by Eurosurveillance and the journal is not responsible for the maintenance of any links or email addresses provided therein.

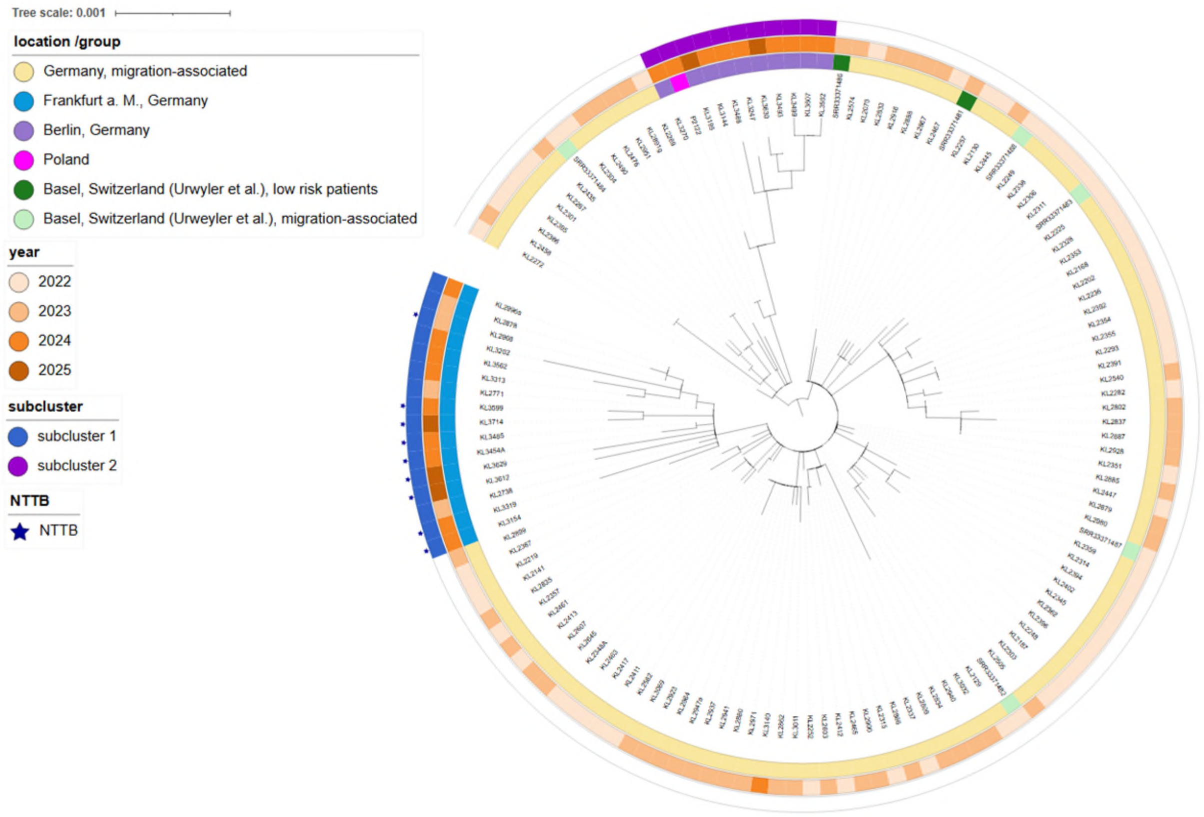


**Supplementary Figure: Maximum Likelihood phylogeny of whole genome SNP-analysis of ST-574 isolates of *Corynebacterium diphtheriae,* 2022–2025 (n = 133) from Germany, Poland and Switzerland, including the two autochthonous sub-clusters.**

*Figure description: Samples are colour-coded based on a combination of geographic location, sub-cluster and date of detection for the respective case, including 99 isolates of newly arriving migrants and the two autochthonous sub-clusters: Sub-cluster 1, including 16 isolates concentrated mainly at the Frankfurt am Main area in blue and sub-cluster 2, including 10 isolates, concentrated mainly at the Berlin area and the Polish isolate (n=11 in total) in purple and pink. ML phylogeny was done with 200 bootstrap replicates and supporting bootstrap percentages (>/= 80%) are indicated at the branches in blue. NTTB isolates are marked with asterisks in dark blue.*

## Supplementary information on resistance and virulence gene prediction:

The profile of detected resistance and virulence genes was uniform among all genomes of the ST-574 cluster. Resistance genes against aminoglycosides (*aph(3'")-Ib*, *aph(3")-Ia*, *aph(6)-I*d), phenicols (*cmx*) and sulfonamides (*sul1*) were present. The virulence gene profile includes typical *C. diphtheriae* adherence factors (*sapD, spaA, spaB, spaC, srtA, DIP_RS14950, DIP_RS17590, DIP_RS19245*) and iron metabolism genes (*ciuA, ciuB, ciuC, ciuD, ciu, fagA, fagB, fagC, fagD, hmuT, hmuU, hmuV, htaA, htaB, irp6A, irp6B, irp6C, DIP_RS20575*).

**Supplementary Methods:**

WGS-based Single Nucleotide Polmorphism (SNP) calling was carried out using snippy [18] and a maximum likelihood tree was built using RaxML-NG v1.2.2 [19] with 200 bootstraps replicates on the WGS data of the 133 ST-574 isolates: Thereof 125 isolates were from Germany (including 99 isolates from the 2022 migration-associated outbreak), one isolate from Poland and seven WGS datasets from Switzerland, published by Urwyler et al. [5]. The Swiss WGS data were retrieved from NCBI Short Read Archive and analysed with the same bioinformatic pipeline. Resistance and virulence gene prediction were performed from WGS assemblies, created with shovill v1.1.0 [20], using abricate v1.0.1 [21] with the Comprehensive Antibiotic Resistance Database (CARD, 2025-Jan-14 ) [22] and the virulence factor database (VFDB, 2025-Jan-14] [23]. Insertions were investigated with ISfinder (https://isfinder.biotoul.fr/blast.php).

**Supplementary Table: Analysed isolates and WGS data with detailed NCBI accession numbers of genomic sequences.**

NTTB isolates are marked with asterisks.

| Sample-ID | Group/location | Year | Sub-cluster | Bioproject accession no. | BioSample accession no. |
|---|---|---|---|---|---|
| KL2070 | migration-associated | 2022 | | PRJNA898270 | SAMN31602461 |
| KL2129 | migration-associated | 2022 | | PRJNA898270 | SAMN31602464 |
| KL2130 | migration-associated | 2022 | | PRJNA898270 | SAMN31602465 |
| KL2141 | migration-associated | 2022 | | PRJNA898270 | SAMN31602468 |
| KL2168 | migration-associated | 2022 | | PRJNA898270 | SAMN31602476 |
| KL2187 | migration-associated | 2022 | | PRJNA898270 | SAMN31602481 |
| KL2202 | migration-associated | 2022 | | PRJNA898270 | SAMN31602487 |
| KL2219 | migration-associated | 2022 | | PRJNA898270 | SAMN31602493 |
| KL2225 | migration-associated | 2022 | | PRJNA898270 | SAMN31602497 |
| KL2236 | migration-associated | 2022 | | PRJNA898270 | SAMN36271205 |
| KL2248 | migration-associated | 2022 | | PRJNA898270 | SAMN36271207 |
| KL2249 | migration-associated | 2022 | | PRJNA898270 | SAMN36271208 |
| KL2252 | migration-associated | 2022 | | PRJNA898270 | SAMN36271209 |
| KL2257 | migration-associated | 2022 | | PRJNA898270 | SAMN36271212 |

| | | | | |
|---|---|---|---|---|
| KL2267 | migration-associated | 2022 | PRJNA898270 | SAMN36271219 |
| KL2272 | migration-associated | 2022 | PRJNA898270 | SAMN36271222 |
| KL2282 | migration-associated | 2022 | PRJNA898270 | SAMN36271224 |
| KL2289 | migration-associated | 2022 | PRJNA898270 | SAMN36271227 |
| KL2293 | migration-associated | 2022 | PRJNA898270 | SAMN36271230 |
| KL2301 | migration-associated | 2022 | PRJNA898270 | SAMN36271233 |
| KL2303 | migration-associated | 2022 | PRJNA898270 | SAMN36271235 |
| KL2304 | migration-associated | 2022 | PRJNA898270 | SAMN36271236 |
| KL2306 | migration-associated | 2022 | PRJNA898270 | SAMN36271238 |
| KL2311 | migration-associated | 2022 | PRJNA898270 | SAMN36271241 |
| KL2314 | migration-associated | 2022 | PRJNA898270 | SAMN36271243 |
| KL2315 | migration-associated | 2022 | PRJNA898270 | SAMN36271244 |
| KL2328 | migration-associated | 2022 | PRJNA898270 | SAMN36271251 |
| KL2337 | migration-associated | 2022 | PRJNA898270 | SAMN36271256 |
| KL2338 | migration-associated | 2022 | PRJNA898270 | SAMN36271257 |
| KL2345 | migration-associated | 2022 | PRJNA898270 | SAMN36271261 |
| KL2348A | migration-associated | 2022 | PRJNA898270 | SAMN36271263 |
| KL2351 | migration-associated | 2022 | PRJNA898270 | SAMN36271264 |
| KL2353 | migration-associated | 2022 | PRJNA898270 | SAMN36271265 |
| KL2354 | migration-associated | 2022 | PRJNA898270 | SAMN36271266 |
| KL2355 | migration-associated | 2022 | PRJNA898270 | SAMN36271267 |
| KL2357 | migration-associated | 2022 | PRJNA898270 | SAMN36271268 |
| KL2359 | migration-associated | 2022 | PRJNA898270 | SAMN36271270 |
| KL2362 | migration-associated | 2022 | PRJNA898270 | SAMN36271272 |

| | | | | |
|---|---|---|---|---|
| KL2386 | migration-associated | 2022 | PRJNA898270 | SAMN36271274 |
| KL2387 | migration-associated | 2022 | PRJNA898270 | SAMN36271275 |
| KL2391 | migration-associated | 2022 | PRJNA1176523 | SAMN49794260 |
| KL2392 | migration-associated | 2022 | PRJNA1176523 | SAMN49794261 |
| KL2394 | migration-associated | 2022 | PRJNA1176523 | SAMN49794262 |
| KL2395 | migration-associated | 2022 | PRJNA1176523 | SAMN49794263 |
| KL2396 | migration-associated | 2022 | PRJNA1176523 | SAMN49794264 |
| KL2402 | migration-associated | 2022 | PRJNA1176523 | SAMN49794265 |
| KL2411 | migration-associated | 2022 | PRJNA1176523 | SAMN49794266 |
| KL2412 | migration-associated | 2022 | PRJNA1176523 | SAMN49794267 |
| KL2413 | migration-associated | 2022 | PRJNA1176523 | SAMN49794268 |
| KL2417 | migration-associated | 2022 | PRJNA1139060 | SAMN42749338 |
| KL2435 | migration-associated | 2023 | PRJNA1176523 | SAMN49794269 |
| KL2445 | migration-associated | 2023 | PRJNA1176523 | SAMN49794270 |
| KL2447 | migration-associated | 2022 | PRJNA1176523 | SAMN49794271 |
| KL2458 | migration-associated | 2023 | PRJNA1176523 | SAMN45815019 |
| KL2461 | migration-associated | 2023 | PRJNA1176523 | SAMN49794272 |
| KL2463 | migration-associated | 2022 | PRJNA1176523 | SAMN49794273 |
| KL2465 | migration-associated | 2023 | PRJNA1176523 | SAMN49794274 |
| KL2467 | migration-associated | 2022 | PRJNA1176523 | SAMN49794275 |
| KL2476 | migration-associated | 2023 | PRJNA1176523 | SAMN49794276 |
| KL2490 | migration-associated | 2023 | PRJNA1176523 | SAMN49794277 |
| KL2505 | migration-associated | 2023 | PRJNA1176523 | SAMN49794278 |
| KL2540 | migration-associated | 2023 | PRJNA1176523 | SAMN49794279 |

| | | | | | |
|---|---|---|---|---|---|
| KL2574 | migration-associated | 2023 | | PRJNA1176523 | SAMN49794280 |
| KL2582 | migration-associated | 2022 | | PRJNA1176523 | SAMN49794281 |
| KL2603 | migration-associated | 2023 | | PRJNA1176523 | SAMN49794282 |
| KL2607 | migration-associated | 2023 | | PRJNA1176523 | SAMN49794283 |
| KL2645 | migration-associated | 2023 | | PRJNA1176523 | SAMN49794284 |
| KL2738 | Frankfurt a. M. | 2023 | 1 | PRJNA1139060 | SAMN42749339 |
| KL2771 | Frankfurt a. M. | 2023 | 1 | PRJNA1139060 | SAMN42749340 |
| KL2802 | migration-associated | 2023 | | PRJNA1176523 | SAMN49794285 |
| KL2809 | migration-associated | 2023 | | PRJNA1176523 | SAMN49794286 |
| KL2833 | migration-associated | 2023 | | PRJNA1176523 | SAMN49794287 |
| KL2834 | migration-associated | 2023 | | PRJNA1176523 | SAMN49794288 |
| KL2835 | migration-associated | 2023 | | PRJNA1176523 | SAMN49794289 |
| KL2837 | migration-associated | 2023 | | PRJNA1176523 | SAMN45815020 |
| KL2862 | migration-associated | 2023 | | PRJNA1176523 | SAMN49794290 |
| KL2864 | migration-associated | 2023 | | PRJNA1176523 | SAMN49794291 |
| KL2866 | migration-associated | 2023 | | PRJNA1176523 | SAMN49794292 |
| KL2867 | migration-associated | 2023 | | PRJNA1176523 | SAMN49794293 |
| KL2878 | Frankfurt a. M. | 2023 | 1 | PRJNA1139060 | SAMN42749341 |
| KL2879 | migration-associated | 2023 | | PRJNA1176523 | SAMN49794294 |
| KL2880 | migration-associated | 2023 | | PRJNA1139060 | SAMN42749342 |
| KL2885 | migration-associated | 2023 | | PRJNA1176523 | SAMN49794295 |
| KL2887 | migration-associated | 2023 | | PRJNA1176523 | SAMN49794296 |
| KL2888 | migration-associated | 2023 | | PRJNA1176523 | SAMN49794297 |
| KL2891g | migration-associated | 2023 | | PRJNA1176523 | SAMN49794298 |

| | | | | | |
|---|---|---|---|---|---|
| KL2899 | migration-associated | 2023 | | PRJNA1176523 | SAMN49794299 |
| KL2900 | migration-associated | 2023 | | PRJNA1176523 | SAMN49794300 |
| KL2916 | migration-associated | 2023 | | PRJNA1176523 | SAMN49794301 |
| KL2923 | migration-associated | 2023 | | PRJNA1176523 | SAMN49794302 |
| KL2928 | migration-associated | 2023 | | PRJNA1176523 | SAMN49794303 |
| KL2937 | migration-associated | 2023 | | PRJNA1176523 | SAMN49794304 |
| KL2940 | migration-associated | 2023 | | PRJNA1176523 | SAMN49794305 |
| KL2941 | migration-associated | 2023 | | PRJNA1176523 | SAMN49794306 |
| KL2947a | migration-associated | 2023 | | PRJNA1176523 | SAMN49794307 |
| KL2951 | migration-associated | 2023 | | PRJNA1176523 | SAMN49794308 |
| KL2968 * | Frankfurt a. M. | 2023 | 1 | PRJNA1139060 | SAMN42749343 |
| KL2971 | migration-associated | 2023 | | PRJNA1176523 | SAMN49794309 |
| KL2980 | migration-associated | 2023 | | PRJNA1176523 | SAMN49794310 |
| KL2996a | Frankfurt a. M. | 2024 | 1 | PRJNA1139060 | SAMN42749344 |
| KL3011 | migration-associated | 2023 | | PRJNA1176523 | SAMN49794311 |
| KL3032 | migration-associated | 2023 | | PRJNA1176523 | SAMN49794312 |
| KL3069 | migration-associated | 2023 | | PRJNA1176523 | SAMN49794313 |
| KL3140 | migration-associated | 2024 | | PRJNA1176523 | SAMN49794314 |
| KL3144 | Berlin | 2024 | 2 | PRJNA1176523 | SAMN44451175 |
| KL3154 * | Frankfurt a. M. | 2024 | 1 | PRJNA1176523 | SAMN49794315 |
| KL3195 | Berlin | 2025 | 2 | PRJNA1176523 | SAMN44451176 |
| KL3202 | Frankfurt a. M. | 2024 | 1 | PRJNA1176523 | SAMN49794316 |
| KL3247 | Berlin | 2024 | 2 | PRJNA1176523 | SAMN49794317 |
| KL3270 | Berlin | 2024 | 2 | PRJNA1197751 | SAMN45814983 |

| | | | | | |
|---|---|---|---|---|---|
| KL3313 | Frankfurt a. M. | 2024 | 1 | PRJNA1176523 | SAMN44451179 |
| KL3319 * | Frankfurt a. M. | 2024 | 1 | PRJNA1176523 | SAMN49794318 |
| KL3454A * | Frankfurt a. M. | 2024 | 1 | PRJNA1176523 | SAMN49794319 |
| KL3485 * | Frankfurt a. M. | 2024 | 1 | PRJNA1176523 | SAMN49794320 |
| KL3488 | Berlin | 2024 | 2 | PRJNA1176523 | SAMN44451177 |
| KL3493 | Berlin | 2024 | 2 | PRJNA1176523 | SAMN44451178 |
| KL3499 | Berlin | 2024 | 2 | PRJNA1176523 | SAMN44405808 |
| KL3502 | Berlin | 2024 | 2 | PRJNA1176523 | SAMN44405809 |
| KL3562 | Frankfurt a. M. | 2024 | 1 | PRJNA1176523 | SAMN49794321 |
| KL3599 * | Frankfurt a. M. | 2024 | 1 | PRJNA1176523 | SAMN49794322 |
| KL3607 | Berlin | 2024 | 2 | PRJNA1176523 | SAMN49794323 |
| KL3612 * | Frankfurt a. M. | 2025 | 1 | PRJNA1176523 | SAMN49794324 |
| KL3629 * | Frankfurt a. M. | 2025 | 1 | PRJNA1176523 | SAMN49794325 |
| KL3630 | Berlin | 2025 | 2 | PRJNA1176523 | SAMN49794326 |
| KL3714 * | Frankfurt a. M. | 2025 | 1 | PRJNA1176523 | SAMN49794327 |
| P2122 | Poland | 2024 | 2 | PRJNA1197751 | SAMN45814984 |
| SRR33371480 | Urwyler et al., low risk patients | 2023 | | PRJNA1123191 | SAMN48197610 |
| SRR33371481 | Urwyler et al., low risk patients | 2023 | | PRJNA1123191 | SAMN48197609 |
| SRR33371482 | Urweyler et al., asylum seekers | 2022 | | PRJNA1123191 | SAMN48197608 |
| SRR33371483 | Urweyler et al., asylum seekers | 2022 | | PRJNA1123191 | SAMN48197607 |
| SRR33371484 | Urweyler et al., asylum seekers | 2022 | | PRJNA1123191 | SAMN48197606 |
| SRR33371487 | Urweyler et al., asylum seekers | 2022 | | PRJNA1123191 | SAMN48197603 |
| SRR33371488 | Urweyler et al., asylum seekers | 2022 | | PRJNA1123191 | SAMN48197602 |
